# An Asymptomatic Large Anterior Sacral Meningocele in a Patient with a History of Gestation: A Case Report with Radiological Findings

**DOI:** 10.1155/2013/842620

**Published:** 2013-12-17

**Authors:** Mehmet Beyazal

**Affiliations:** Department of Radiology, School of Medicine, Yüzüncü Yıl University, 65080 Van, Turkey

## Abstract

Anterior sacral meningocele is characterized by herniation of the meningeal sac due to a developmental bone defect in the front of a sacrum bone. It was first described in 1837. The sacral meningocele may be congenital or acquired. It is usually discovered during a rectal or pelvic examination as a cystic lesion or discovered incidentally. Most of the symptoms are due to compression on the adjacent organs. In this paper, we present a case of an asymptomatic female patient who had a pelvic cyst detected during a routine obstetric ultrasound examination. We show radiological findings of the detailed postpartum evaluation of the cyst, which led to detection of sacral agenesis, huge anterior sacral meningocele, and significant arcuate uterus.

## 1. Introduction

The anterior sacral meningocele is defined as herniation of the meningeal sac due to developmental bone defect in the front of a sacrum bone [1]. It is reported that anterior sacral meningocele had been first described in 1837 [2, 3]. It may be congenital or acquired. While anterior sacral meningocele usually occurs as a congenital defect, most acquired sacral meningoceles occur due to dural ectasia associated with neurofibromatosis, Marfan's syndrome, and Ehlers-Danlos syndrome [4]. Most of the symptoms are due to compression on the adjacent organs, and the most common symptom is constipation [5].

In this article we present the radiological findings of sacral agenesis, large anterior sacral meningocele, and bicornuate uterus in the case of an asymptomatic female patient with a history of gestation.

## 2. Case Report

**Figure 1:**
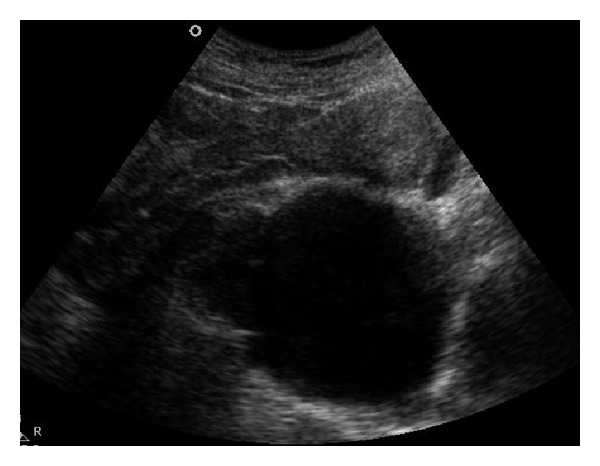
Sagittal ultrasound image of the pelvic area showing a thin-walled cystic lesion compressing the uterus on the posterior.

**Figure 2:**
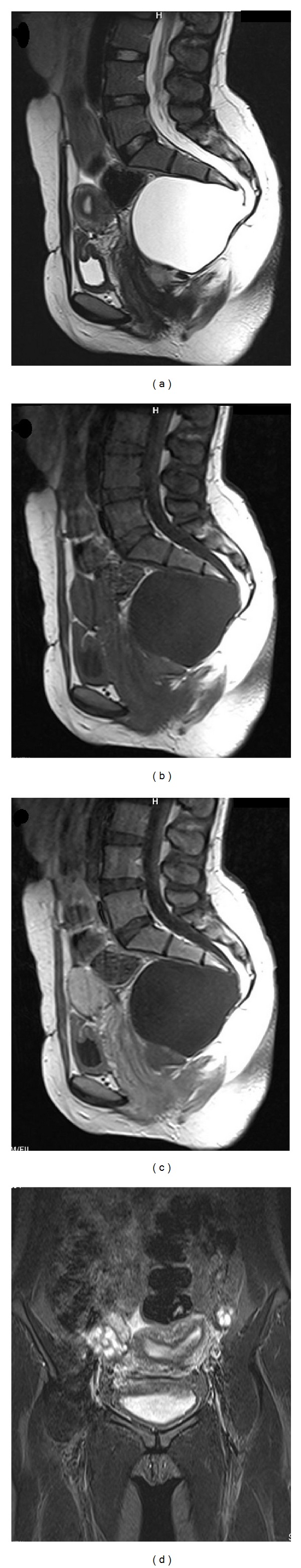
Sagittal T2-weighted MRI images of the thin-walled hyperintensity in the posterior of the uterus and the rectum (a) and hypointense cystic lesion on T1-weighted images (b). The 4th and 5th sacral bones are not seen. In the posterior of the cyst, communication with spinal subarachnoid space is present. The contrast enhancement is not observed in sagittal postcontrast T1-weighted images (c). Conus medullaris ends at the level of the 4th lumbar spine. The distinct arcuate uterus is seen in the coronal T2-weighted images (d).

About 6 months before at 22 weeks of gestation, a cystic lesion was identified during a routine obstetric ultrasound examination of the pelvic area in a 23-year-old female patient without any previous discomfort. Due to the compression of the cyst on the cervix, the delivery was achieved by caesarean section. Postpartum ultrasonographic examination showed that there was a 7.5 × 8.5 cm sized thin-walled cystic lesion in the midline of the pelvic region compressing the rectum, uterus and bladder (Figure 1). The origin of the lesion could not be determined during the ultrasonographic examination. The T2-weighted magnetic resonance imaging (MRI) showed a thin-walled hyperintensity in the uterus, and the rectum posterior while T1-weighted images revealed a hypointense cystic lesion. The cyst was not associated with the ovaries. The fourth and fifth sacral bones were not seen. In the posterior, the cyst was communicating with the spinal subarachnoid space (Figure 2). Based on these findings, the patient was diagnosed with sacral agenesis and anterior sacral meningocele. In addition, MRI showed that the conus medullaris ended at the 4th lumbar spine level and a distinct arcuate uterus was observed. The patient's neurological examination was normal. Complete blood count and serum biochemical tests were within normal limits.

Because she planned another pregnancy, gynecological surgeons and neurosurgeons suggested to the patient a surgical management. But she denied.

## 3. Discussion

The anterior sacral meningocele is an anterior sacral defect or a herniation of the meningeal sac from the sacral foramen to the anterior. The congenital form of the sacral anomaly can vary from minor sacral defects to complete sacral agenesis. The anterior sacral meningocele may be isolated or may be associated with other congenital abnormalities. Urogenital tract and uterine anomalies, anorectal malformations, lipomas, sacrococcygeal teratoma, epidermoid tumor, and dermoid cysts may accompany the anterior sacral meningocele [4, 6]. The anorectal anomaly, ventral sacral defect, and presacral mass are known together as “Currarino's triad.” The presacral mass meningocele, teratoma, or enteric cyst might be in “Currarino's triad” [7].

The anterior sacral meningoceles account for approximately 5% of retrorectal masses. They are generally diagnosed in the second or third decades and are more prevalent in women. They may be asymptomatic or present as nonspecific symptoms such as long-term constipation, urinary dysfunction, dysmenorrhea, lower back pain, or perineal hypoalgesia [1, 4, 5]. These symptoms may be due to direct compression of the herniated meningeal sac, spinal cord tethering, or sacral nerve root compression. In addition, congenital defects that may occur in autonomic innervation of the bladder and anal sphincter may be associated with constipation and urinary dysfunction. Symptoms such as dysmenorrhea and dyspareunia may cause direct compression as well as lead to venous congestion as a result of the compression on the pelvic veins [4, 5]. In addition, headache can be seen in 10 to 15 percent of patients. The changes in the intra-abdominal pressure are thought to occur as a result of effects of intracranial pressure via the meningocele pouch [3, 4]. Our the patient was asymptomatic and pelvic cyst that was detected during a routine obstetric ultrasound examination was then diagnosed postpartum by MRI evaluation.

Ultrasonography, computed tomography, MRI, and myelography tests can be used for radiological evaluation. Ultrasonography can be used for the first imaging evaluation of pelvic lesion, and, if necessary, can also be successfully used in followups. However, as was the case with our patient, the origin of the pelvic cystic lesion cannot always be detected with ultrasound. Computed tomography is an important tool in providing detailed information about bone lesions or abnormalities [1]. MRI provides high soft tissue contrast; is capable of multiplanar imaging; and is a fast, non-invasive, and reliable imaging technique. It can detect communication between the anterior sacral meningocele and the spinal subarachnoid space as well as provide detailed information about accompanying pathologies such as spinal cord tethering or a tumor [4]. However, in some cases where the communication with the subarachnoid space is narrow, the MRI may fail to demonstrate that communication. In such cases, there might be a need for a myelography examination after a intrathecal contrast injection, which can be applied under fluoroscopy or as a computed tomography myelography [2].

In addition, cystic lesions that are located in the presacral region should be considered during the diagnosis. These may include gastrointestinal and genitourinary tract tumors, dermoid and epidermoid cysts, aneurysmal bone cyst, hamartoma, intrasacral meningocele, hydatid cyst, lipoma, lymphangioma, perineurial cyst, rectal duplication, reproductive tract tumors, teratomas, and teratocarcinoma [4, 8]. Moreover, the most important finding in the differential diagnosis is to demonstrate the communication between the cystic lesion and subarachnoid space [6].

The anterior sacral meningocele does not regress [2]. Although it may be asymptomatic, some authors have reported infectious complications such as meningitis. Therefore, surgical treatment is recommended [5, 9]. Some physicians have recommended surgical treatment if there is an increase in lesion size or if the sacral meningocele is symptomatic [2].

## 4. Conclusion

Since the anterior sacral meningocele is a rare entity, it may be confused with more common cystic lesions of gynecologic origin during routine ultrasound examinations of female patients. When cystic lesions of a presacral location whose relationship with gynecological organs is not clear are detected during an ultrasonographic examination, an MRI examination would be an appropriate approach for evaluation an anterior sacral meningocele.
